# Evidence-Based Medicinal Plants for Modern Chronic Diseases

**DOI:** 10.1155/2014/948385

**Published:** 2014-04-16

**Authors:** Yong-Ouk You, James William Daily, Tong Ho Kang, Young-Rae Lee, Serkan Selli

**Affiliations:** ^1^Department of Oral Biochemistry, School of Dentistry & Wonkwang Research Institute for Food Industry, Wonkwang University, Iksan 570-749, Republic of Korea; ^2^Research & Development, Daily Manufacturing, Inc., Rockwell, NC 28138, USA; ^3^Department of Oriental Medicinal Materials & Processing, College of Life Sciences, Kyung Hee University, Global Campus, Gyeonggi 446-701, Republic of Korea; ^4^Center for Metabolic Function Regulation, Wonkwang University School of Medicine, Iksan 570-749, Republic of Korea; ^5^Department of Food Engineering, Faculty of Agriculture, University of Cukurova, 01330 Adana, Turkey

Plants have been used as medicines throughout recorded human history by most, if not all, ethnicities and cultures. The knowledge of how to use plants for medicine has been passed down through generations by many means including word of mouth and ancient pharmacopoeias. Modern science has devoted considerable research to characterizing the efficacy and mechanisms of action of many medicinal plants, but this remains an area of vast research potential. This special issue of Evidence-Based Complementary and Alternative Medicine is devoted to publishing the most important recent scientific advances in describing the efficacy, safety, and mechanisms of action of medicinal plants for modern chronic diseases.

Global environmental and human lifestyle changes have dramatically increased the incidence of chronic diseases in the 21th century. Modern chronic diseases cause great human suffering, health care expenses, and lost productivity despite the advances of modern medicine. Evidence-based medicinal plants have great potential as safe and effective alternative medicines for modern chronic diseases. Although medicinal plants are an established part of complementary and alternative medicine with efficacy supported by thousands of years of clinical experiences in treating human diseases, many lack scientific support, since medicinal plants are the products of experience-based medicine. Nevertheless, numerous scientific experiments support the efficacy of medicinal plants.

In this special issue, 3 review articles and 11 research articles were published addressing the safety and efficacy of evidence-based medicinal plants for treating modern chronic diseases, such as diabetes, hypercholesterolemia, obesity, major depressive disorder, chronic infection by antibiotic resistant bacteria, postmenopausal osteoporosis, rheumatoid arthritis, allergy, asthma, chronic inflammation, and myasthenia gravis. These studies include clinical trials with rigorous statistical analyses, phytochemical, pharmacological, and toxicological activity tests in animal models and cell-based studies, analytical characterization of bioactive components in medicinal plants, and investigation of action mechanisms of medicinal plants, all of which are essential for complementary and alternative medicine to have a solid evidence-based foundation.

*Yong-Ouk You* *Yong-Ouk You* *James William Daily III* *James William Daily III* *Tong Ho Kang* *Tong Ho Kang* *Young-Rae Lee* *Young-Rae Lee* *Serkan Selli* *Serkan Selli*

